# Nutraceuticals Prepared with Specific Strains of Probiotics for Supplementing Gut Microbiota in Hosts Allergic to Certain Foods or Their Additives

**DOI:** 10.3390/nu15132979

**Published:** 2023-06-30

**Authors:** Divakar Dahiya, Poonam Singh Nigam

**Affiliations:** 1 Wexham Park Hospital, Wexham Street, Slough SL2 4HL, UK; 2 Biomedical Sciences Research Institute, Ulster University, Coleraine BT52 1SA, UK

**Keywords:** probiotic, bacteria, fermentation, synbiotic, food, nutrition, gut, lactose, inflammation, plant-derived food

## Abstract

Certain nutrients cause discomfort, sensitivity reaction, and an intolerance for certain foods or their ingredients when ingested by some consumers. Food reactions and gut inflammation-related problems are increasing worldwide. The primary form of management would be the avoidance of such foods, followed by treatment of their symptoms. Adopting a nutritional–therapeutic approach and establishing practices for the inclusion of functional foods and nutraceuticals in the diet could improve the ecology of gut microbiota and alleviate inflammation in the GIT. For this purpose, specific species of microorganisms characterized as probiotic strains have been studied to produce functional food and fermented beverage products. Commercially sold, such items are labelled as probiotic products, displaying the name/s of strain/s and the viable numbers of them contained in the portion size of the products. The importance of the growth of probiotic functional foods is that they can be consumed as a source of nutrition and their intake helps in the subsistence and recuperation of friendly gut bacteria. Probiotics have been reported for their role in ameliorating the risk of food reactions. Probiotic administration has been implemented for its role as an auxiliary improvement and for the prevention of food sensitivities common among pediatric patients. Probiotic products based on non-dairy substrates have potential as nutraceuticals for lactose intolerant consumers who are allergic to dairy milk products. Therefore, the aim of this article is to review GRAS microbial species characterized as probiotics up to the level of their specific strain’s name and/or number. These have been used to produce nutraceuticals that are sources of beneficial bacteria for easing discomfort and allergic reactions by maintaining an inflammation-free gut.

## 1. Introduction

In the last few decades, food intolerance has become an important global health concern. Food-related health disorders are rising, alarming, and affect about 10% of the global population’s quality of life: the most affected group being young children [1]. Most industrialized nations have a higher occurrence of food sensitivity issues than developing countries, though the frequency in these regions is also increasing [2]. The expansion of food intolerance is a multifaceted phenomenon that is regulated principally by individuals’ immune responses, gut–epithelial function, genetics, and other environmental influences [3]. Sensitive reactions to some food ingredients (described in further sections) are primarily caused by a disturbed balance in the microbiota of the gastrointestinal tract [4]. The epidemiology of food varies by age group and the geographical setting of consumers. Children usually have higher rates of food sensitivity compared to adults for a few reasons [5], and this has been discussed in Section 2.2. Although certain foods act as allergens that are specific to a particular geographical area, for example, an allergic reaction to peanuts being very common in Western countries, a shellfish allergy is frequently noticed in populations residing in Asian countries due to the high level of tropomyosin found in shellfish. However, regardless of the region of habitation, there are some common foods causing discomforts, including lactose-containing, dairy-sourced milk and its products, eggs, and gluten-containing cereals, such as wheat [6]. 

This article focuses on incorporating probiotics into diet as a means of sustaining gut health based on studies published in recent decades. The information presented in the following sections mainly deals with the types of food which have been identified as causes of discomfort and intolerance, along with the symptoms of sensitivity reactions experienced by some consumers to certain foods. References to studies conducted on specific strains of probiotic microbial cultures have been included. These purposely selected strains have been utilized in the production of functional foods, some commercial probiotic food products, and supplements that are readily available to consumers online or in shops. Their intake has been suggested as useful for sustaining beneficial gut microbiota in consumers and for the restoration of disturbed gut microbiota in patients suffering from a condition of dysbiosis. A balanced gut microbiota benefits from this nutraceutical therapeutic approach and can have an active role in reducing food-related discomfort. 

The objective of this article is not to provide the clinical biochemistry of allergic reactions, pathogenicity of dietary diseases, or their medical treatment, but to review GRAS microbial species characterized as probiotics up to the level of their specific strain’s name and/or number. 

## 2. Foods Causing Intolerance Reactions

Food allergy (FA) is the reaction of the body after the consumption of a specific food or certain components and ingredients, including preservatives, additives, and coloring materials used in the preparation of food and beverage products [7]. FA is defined as “An adverse health effect arising from a specific immune response that occurs reproducibly on exposure to a given food” [8]. Allergens are specific elements of a food item identified by the consumer’s immune system, which causes symptoms of food-specific intolerances. Allergies can show different reactions, involving effects ranging from very moderate and temporary to serious anaphylactic responses, for example, the reactions caused by nuts, which is one of the most familiar food allergens. Eating peanuts or a product containing traces of peanuts could cause a very serious reaction in some people. A real allergy affects the immune system, and in particular, an immunoglobulin termed, IgE (antibody), is involved in the condition of anaphylaxis [9].

For some allergy sufferers, symptoms are invisible and sometimes have life-threatening consequences that can affect their lives on an everyday basis. The list of food items that might cause allergic reactions in some consumers can be organized into two categories based on their sources. The first category includes plant-based items, such as gluten-containing cereals, mustard, peanuts, sesame seeds, soybeans, and lupin present in a wide range of food products prepared from lupini beans, including pasta or noodles, sauces, and baked goods (such as bread, pastries, and pies). The other category contains those items which have been sourced from animals, including crustaceans (prawns, crabs, lobsters, shrimp, and krill etc.), eggs, milk, molluscs (clams, scallops, oysters, octopus and squid, etc.), and fish [6]. Even though most foods presented in these two categories might act as allergens for some, they are generally safe for people who are not allergic to such items. In current general practice, to comply with food safety and health regulations, food items have all their ingredients listed (specifically mentioning all probable allergens), whether they are used in ready meal products or in the preparation of menus for catering and dining services.

The primary variation between an intolerance for a type of food and an allergy is that while intolerance for certain foods can be very disagreeable, a food allergy could be life-threatening for some consumers. An allergy usually appears as an immediate swelling on the face or causes a restriction of the airways. Even if the FA is initiated by a very small amount of the triggering element contained in an item of food, an urgent condition of FA will require medical attention. However, an intolerance to a particular food is realized as a gradual response, mostly revealing unpleasant digestive indications, and is usually experienced after exposure to a substantial amount of the reaction-triggering item acting as an allergen [8]. Table 1 summarizes food types that could be potential allergens causing symptoms of intolerance.

### 2.1. Symptoms of Allergies and Discomforts Caused by Food

Certain FAs can present much more serious and severe symptoms, such as breathing shortness, swollen mouth, face and throat, skin rashes and difficulty in swallowing [8]. The most serious and potentially fatal allergic reaction is anaphylaxis. Other allergic reactions include digestive indicators such as reflux, vomiting, abdominal ache, problem in swallowing certain foods or liquids (dysphagia), diarrhea, growth failure, and feeding disorders. The symptoms might also show up as cutaneous manifestations, such as raised, itchy rashes on the skin (Urticaria), swelling of the deeper layers of the skin caused by a build-up of fluid, mostly affecting the area around the eyes and lips (Angioedema), flushing, itchy skin with an irritating scratching sensation (Pruritus), or eczema. Some other symptoms could be respiratory indicators such as wheezing, breathlessness, an intense tightening in the chest or a feeling of suffocation (Dyspnea), nasal congestion, sneezing, clear nasal discharge (Rhinorrhea), or the inflammation of nasal tissues (Rhinitis) [10]. 

FAs have been mainly correlated with alterations in the permeability of the intestinal mucosal layer, which embodies the main course of pathogenesis [11], and might produce some of the illness symptoms, such as diarrhea. This change can compromise the barrier functionality of epithelium, which affects important activities of the intestine, for example, the assimilation of digested food and the absorption of nutrients. In some cases, the symptoms can include digestive indications, such as stomach cramps or diarrhea, and some people could suffer from skin ailments like severe rashes. A known example of such a condition is an intolerance to gluten. Sufferers of a gluten allergy (celiac disease) experience a range of symptoms involving discomfort in the digestive system, painful joints and fatigue or even drowsiness after eating gluten-containing food items (a personal experience for years) [12].

### 2.2. Imbalanced Gut Microbiota Cause of Food Discomforts and Allergies

Gut microbes and their metabolites are actively involved in the development and regulation of host immunity, which is an important requirement for avoiding the risk of food allergies. Therefore, some recent studies have focused on the gut microbiota–immune axis [4,7,9]. The normal gut microbiota could be disturbed due to several reasons that may enhance the risk of emerging allergies and other conditions of discomfort. The use of antibiotics to treat gastric infections caused by the intake of contaminated food and water, could be the reason for a disturbance in normal gut microbiota [13], which in turn might increase the possibility of allergic disorders that have become more common in children [14]. The allergies triggered by the ingestion of specific foodstuffs are now considered pediatric diseases. The symptoms of a weakened gut system could be disturbances in sleep, dermal irritations, intolerances, and unintentional changes in body weight [15]. 

In particular, more and more children suffer from one or another kind of allergy. A report has suggested that babies delivered via a caesarean section have a higher possibility of developing allergies in their adult lives [16]. Often associated with the increased use of antibiotics in infants for the treatment of common ailments (ear infections and tonsillitis), infants often fail to build a sustainable population of normal gut flora, which is needed to maintain strong immunity throughout one’s life. Antibiotic treatments in the first year of a child’s life have shown the possibility of developing an allergy by up to 50% [10,12]. Considering the fact that the most valuable gut flora is initially passed on to newborns from their mothers by the way of the birth canal via the natural delivery of a baby, the tendency of having a weaker and imbalanced gut microbiota could be found in the family history of disturbed gut health. Research suggests that supporting gut health, by the way of consuming live probiotic cultures containing nutraceuticals, helps to prevent suspected sensitivity to allergens and their symptoms, such as skin rashes and eczema [17,18].

## 3. Role of Probiotics in Restoring Gut Health

The evidence for a connection between gut microbiota and allergies comes from the fact that people suffering from several allergies had a reduced diversity of bacteria in their gut microbiota. In persons having an allergy to a single food item, the gut microbiota was detected to be affected by peanuts more than any other type of food. In fact, an individual that is sensitive to more allergens had an imbalanced gut microbiota with a lower population of beneficial gut bacteria. Researchers were able to detect that these persons had lower levels of Clostridiales bacteria and increased levels of Bacteroidales. The report concluded that the intervention of gut microbiota through the use of probiotics may benefit the reduction or prevention of some allergies [19]. Studies have led to the detection of various biomarkers, involving basophil, T cells and Igs, which are specific to allergens and the microbiome of host [20,21,22]. Most allergic disorders are indicated by a type 2 immune response relating to eosinophils, Th2 cells, lymphoid cells, mast cells, and M2 macrophages [23,24]. 

### 3.1. Probiotic Strains as Biotherapeutic Agents

The consumption of probiotic food, beverages or synbiotic products helps to improve the immune system by supplementing gut microflora, which is particularly beneficial for a population suffering with allergies to build up the lost diversity of their gut bacteria. Supplementing a normal diet with probiotic foods would help in the repair of reduced gut diversity, and through this gastronomical way, a healthy gut could be beneficial to sufferers of FAs. Due to the fact our gastrointestinal tract is the site for 70% of immune cells, the gut microbiome works together with the immune system, as well as it can, to also help in the modulation of its responses [25]. 

In addition to the improvement in organoleptic properties and nutritional quality of foods, it is generally accepted that the food-fermentation process also contributes in the maintenance of gut health by enriching foods with an active population of probiotic cultures [26]. The well-known probiotic effect is likely achieved through the comfort of eased lactose-intolerance symptoms by the ingestion of lactose-free fermented milk and bio-yogurt. In the fermentation process performed by suitable probiotics, the lactose contained in dairy-sourced milk is utilized by lactic acid bacteria and converted into lactic acid [27]. The other health benefits of the consumption of fermented milk products have been linked with the decline in adiposity factors, together with correcting the body mass index [28], type-2 *Diabetes mellitus* [29], and cardiovascular diseases [30]. Moreover, the randomized control trials have also studied the impacts of fermented products including a probiotic beverage-kefir [31], fermented mixed vegetables and kimchi [32], lacto-fermented cabbage for sauerkraut [33], natto [34], and sourdough bread [35]. 

Concerning the mechanism of beneficial properties, the bacterial metabolism during food fermentation [26] reduces the content of high-calorie polysaccharides, improves carbohydrate tolerance, increases the digestibility of fermented carbohydrates, and reduces the concentration of non-nutritive components usually present in raw unfermented materials. The fermented foods act as synbiotic products and increase the availability of bioactive molecules, vitamins, amino acids, organic acids and cofactors, which are the metabolites synthesized by probiotic strains. The bioactive molecules include flavonoids, ɣ-aminobutyric acid, linoleic acid or enzyme inhibitors. Furthermore, the human immune system is closely linked to the gastrointestinal tract and, therefore, the probiotic bacteria contained in fermented foods are expected to play an immunomodulatory function [31,32,33,34,35]. Hence, probiotic strains have been studied for their capability to regulate inflammation in the intestinal epithelial and the activity of immune cells. For example, a study was conducted to assess if cheese consumption at the age of 18 months has a protective effect against allergic diseases during a child’s first six years of life. The report stated the influence of fermented foods for reducing the risk of childhood allergies [36].

### 3.2. Specific Probiotic Strains Used to Prepare Functional Food

In a general practice to conduct traditional food fermentations, bacterial starters have been used mainly for their ability to ferment a variety of raw materials, even though the probiotic quality of cultures was not proven. From the perspective of the preparation of nutraceuticals, the purpose is defined as a way to produce a quality functional food for health improvement, as well as sustaining well-being. Therefore, it is important to combine the beneficial characteristics of bacterial cultures’ potential and selectively employ them in food production for their probiotic activities [26,30,32]. Nevertheless, this criterion is mainly dependent on specific strains of probiotic cultures. Research demonstrates that the benefits of probiotics are seen at the level of their strains, i.e., one strain of the same species may help with gut health, while another strain of same species might be useful for the control of antibiotic-associated diarrhea. The gut-friendly bacteria could have a positive effect on the immune system, simply by aiding to improve the diversity of gut microbiota [13]. An example is NCFM^®^, which is an extensively-studied strain of *Lactobacillus acidophilus* used in clinical studies. It was provided to the sufferers of rhinitis, which is an allergy caused by birch pollen [37]. Another report indicated the effectiveness of a strain characterized as CGMCC of bacteria *L. rhamnosus* for easing the peanut allergy, and a different strain, GG^®^ of *L. rhamnosus*, was used to ease eczema and other atopic conditions [38]. Therefore, for the effective application of appropriate, specific species of probiotic microorganisms in nutraceuticals, their identification and characterization at strain number/name level are essential [26] (Table 2). 

## 4. Probiotic Nutraceutical Products 

Consumers suffering with different food allergy problems have started looking for new, natural and safer options in the form of health-food products [44]. Viewing this requirement, several studies have been performed where functional food and beverages can be prepared in a controlled process of fermentation using selected probiotic strains, which are presented in Table 2. These strains are employed in the fermentation of either dairy-based or non-dairy-based, agriculture-sourced substrates. Alternatively, several nutraceuticals have been commercially formulated to meet the need of consumers for the solution of specific health issues. Such symbiotic products are designed with the supplementation of selected probiotic strains, alongside the appropriate materials derived from plant sources, known as prebiotics [26]. 

Probiotic food products may help reduce bloating and flatulence in people suffering from irritable bowel syndrome (IBS) [50]. A strain of *Lactobacillus rhamnosus* GG supplemented with extensively hydrolyzed casein formula was studied to reduce the incidence of other allergic indications in children suffering from an intolerance to cow’s milk [51,59,60]. The fermented products have been effective as synbiotics for their dual effect, and are beneficial for allergy-free and healthy nutrition, and to relieve gastrointestinal tract inflammation, IBD, and IBS, and also in preventing the induction of cancer [61,62]. Innovative products prepared using fewer preservatives and chemical coloring agents (Table 1), with the requirement of minimal processing, are the focus of the study conducted by the research and development team of the food industry.

### Lactose-Free Products for Dairy-Allergic Population

The allergy related to lactose intolerance in a big population of consumers has initiated the development of dairy-free products using plant-sourced substrates, or a combination of materials obtained from dairy and plant, or two plant-sourced ingredients. The purpose of using different combinations of materials is for adding variation in the composition of plant materials for their carbohydrate, protein and lipid contents [63]. Traditionally, probiotic bacteria have been primarily isolated from the products prepared from dairy-milk, therefore the efficient sources for delivering probiotic bacteria in the gut are fermented products based on dairy, such as yogurt, fermented-milk, and kefir, which is a fermented milk-based beverage [64,65]. However, non-dairy-based functional foods are being considered as appropriate carriers of probiotic cultures. These would aid the percentage of the global population who suffer from lactose intolerance, items containing dairy milk, and those who have difficulties with the digestibility of dairy-based products. In addition, some customers are increasingly buying dairy-free and non-animal products, which are produced from economical dairy alternatives, mainly plant-sourced materials like cereals, grains, and vegetables; thus, these materials are being used in food fermentation for the production of nutraceutical and functional foods [66,67]. 

Raw materials sourced from plants also offer a favorable environment that safeguards the sustainability of probiotic cultures during the shelf-life of a product. The health complications and alternative dietary options for vegans have supported a requirement for dairy-free foods, for instance, fermented cereals and vegetables, and plant-based milk as alternatives [66]. Consequently, there has been an ever-increasing demand for vegan products, which can be produced for people with dietary limitations [67]. The recognized benefits of plant-based products as suitable options providing allergy-free nutrition and health benefits for vegans, present these products with the possibility of their commercial production for global market [68]. Probiotic strains have been selected for their ability to ferment carbohydrates and proteins that are present in dairy-alternative milk obtained from soya, oats, almond and coconut, etc. Studies on such plant-based fermented products, like probiotic beverages and yogurts, have considered the growing occurrence of allergies and gut inflammation [69]. 

However, the raw materials obtained from plant sources should be first analyzed for their constituents for instance allergens, phytic acid, tryptic-inhibitors, alkaloids and carbohydrates [70]. A moderate conversion of these resources for their desired qualities, i.e., nutritional, organoleptic and health benefits, will also be dependent on the use of specific probiotic cultures in the fermentation process. The raw materials, processed for solid or liquid food, can be fermented by appropriately selected and efficient probiotic strains (Table 2). This offers the availability of nutritional options with a variety of wide-reaching, probiotic products for consumers of different requirements. Economical and seasonal geographically available plant resources, include cereals and grains (oat, barley, rye, sorghum, rice, wheat, millet), legumes (soya bean, lentils, peas, faba bean, lupini), and leafy and root vegetables (cabbage, gherkins, cucumber, carrot, beetroot, onions, cassava, manioc, etc.) [71,72].

For the production of commercial nutraceuticals, in order to select a particular probiotic strain as an active and safe starter for fermentation of different substrates, its immunomodulatory properties are considered as screening benchmarks (Table 3).

## 5. Intervention of Probiotics for Normalizing Food Discomforts 

**Figure 1:**
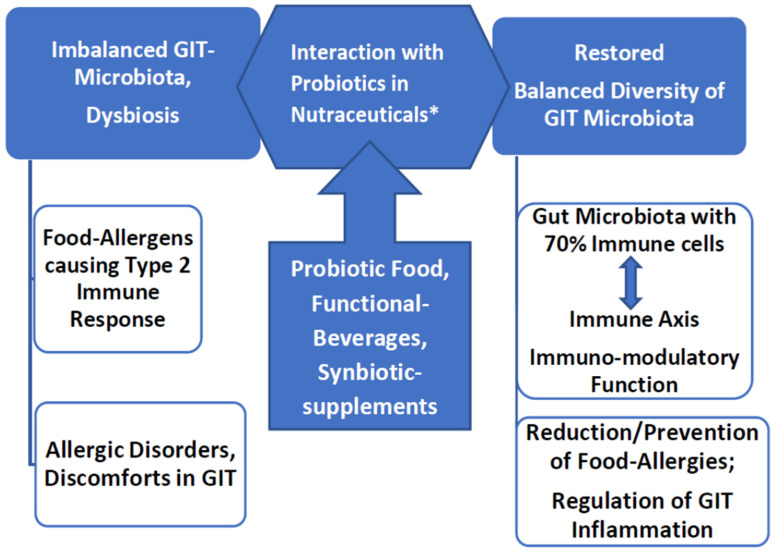
Allergies and discomforts caused by certain foods could be relieved by Probiotic strains incorporated as therapeutic agents in synbiotic nutraceuticals, supporting the gut microbiota-immune axis. * Nutraceuticals include food and beverage products produced via the fermentation process, completed with the activity of specific microbial strains that have been characterized as probiotics. For some consumers’ preference for alternatives over fermented foods, formulated preparations of synbiotics (Prebiotics mixed with probiotics) and commercial supplements are available. GIT-Gastrointestinal Tract [Figure drawn by authors D.D., P.S.N.].

The strategy of employing probiotic cultures is important for the expansion of immuno-modulatory functional foods. Fermented foods, including natural yogurt and probiotic beverages like kefir, have been proven in several research studies to relieve food allergies, and several health issues and discomforts [91]. These products may be appropriate in the situation of a compromised intestinal epithelium in consumers with different food allergies, or at the early stages of inflammation in their digestive tract [92,93]. The probiotic strains are known to synthesize bioactive molecules that are useful for consumers’ health [94]. With this in mind, it would rationalize these probiotic products to be considered as nutraceuticals (Figure 1).

The allergic reaction, which is an inflammatory response, is mainly induced by Th2 [95]. An imbalance between regulatory T cells (Treg) and Th17 in T-lymphocyte subsets also leads to immune disorders [96]. The therapeutic effect of *Bifidobacterium lactis* has been reported for its amelioration of the risk of developing a food allergy in children by affecting a relative percentage of Treg and Th17 cells. A group of 158 children with food allergies (BG) were given a 10 mL probiotic solution of *B. lactis* (1 × 10^6^/mL) daily for three months, and the placebo group of 158 children with food allergies (CG) received a control solution containing no probiotic culture. A mouse allergy model was treated with *B. lactis* which was established by shrimp tropomyosin. The measured data included allergic symptoms, serum IgE, and food antigen-specific IgE. Relative mRNA levels of cytokines associated with Treg- and Th17- were measured by the quantitative PCR method. Researchers reported a remarkable reduction in allergic symptoms, serum levels of IgE and food antigen-specific IgE of group-BG after a three-month therapy, compared to group-CG. Similar results were obtained in the mouse allergy model. The intervention study with *B. lactis* increased the ratio of Treg and Th17 cells; the relative mRNA levels of FoxP3 and TGF-β associated with Treg cells were found to be higher, whereas relative mRNA levels of IL-17A and IL-23 associated with Th17 were reduced. The probiotics intervention studies reported an increased ratio of Treg/Th17 cells, which was the main factor involved to suppress the occurrence of allergic reactions [97]. Several intervention studies in clinical trials have proved the effectiveness of probiotics in relieving gut-related disorders and discomforts caused by certain foods [18,33,40,41,46,47,51,56].

## 6. Conclusions and Future Perspectives

A global occurrence of allergic diseases has presented a considerable medical and socio-economic problem in recent years. Studies have shown evidence that allergies are a result of imbalanced diets, irregular lifestyles, and environmental factors, which have become the common causes. Nutrition comprising processed foods, together with a stressful routine and less exposure to a natural, open environment, limits our contact with naturally occurring beneficial bacteria. These are some of the contributing factors in the development of allergies due to the reduced immunity in the absence of a well-established gut microbiome. A combination of factors may influence the diversity of lactic acid bacteria in the gut and, as a result, our immune systems are affected, thus becoming more sensitive to food allergens.

The future perspective includes the analysis of the results obtained in studies conducted on the development of gut microbiota during infancy, and observing the impact of introducing allergenic foods in infants. This is because gut microbiota plays an important role in the immunological training of the hosts, starting in the early years of their development. The gut microbiota of infants might be developed during their growth by the randomized introduction of allergen foods alongside breastfeeding, which would help in the maturation and to establish the gut microbial communities [98,99].

The application of only characterized individual strains or consortia of probiotic bacteria can be studied to renovate nutritive food matrices through fermentation into synbiotic products. Functional food or beverages should be fortified with naturally available and safer bioactive compounds that are extracted from edible plant sources for flavor enhancements, coloring materials, and as antimicrobial agents for the preservation of food or beverage products, instead of the addition of chemical compounds, which might cause allergic reactions [100,101,102]. The designed supplements of specifically selected probiotic strains as commercial products would provide consumer-friendly therapeutic options to minimize gut disorders and discomforts caused by certain foods. The synbiotics help in reducing the inflammation in the intestinal epithelium, stressed by pro-inflammatory lipopolysaccharides from allergy-causing molecules that are present in certain food items. These functional products should be further assessed in pre-clinical studies of digestive disorders, particularly allergic reactions to specific foods like nuts, dairy products, and ingredients sourced from animals or seafood. These items will offer more nutraceutical options to currently available products for vegans and vegetarians suffering from lactose allergies.

## Figures and Tables

**Table 1:** Foods and ingredients that might cause discomfort and trigger allergic reactions in some consumers *.

Category of foods, Ingredients, Additives	Items as Potential Allergens
Plant-based products	Gluten-containing cereals, Nuts (peanuts, walnuts, almonds, hazelnuts, pecans, cashews, pistachios and Brazil nut), mustard, sesame seeds items, soybeans, food products prepared from lupini beans (pasta, noodles, sauces, bread, pastries, pies)
Food sourced from sea	Crustaceans (prawns, crabs, lobsters, shrimp, and krill etc.), Molluscs (clams, scallops, oysters, octopus and squid etc.)
Dairy-based Products	Lactose-containing dairy milk, Products prepared using dairy milk
Preservatives, chemicals added in food for antimicrobial activity to extend shelf-life	Benzoic acid (E210), and its sodium, potassium and calcium salts (E211–213); Parabens, Sulphites, Nitrites, Nitrates, Acetic Acid BHA (butylated hydroxyanisole), BHT (butylated hydroxytoluene)
Flavor Enhancers, Sweetener-Additives	Monosodium Glutamate (MSG), Hydrolyzed vegetable protein, Aspartame, High Fructose Syrup
Colorings used in processed food, beverages, snacks and candies	Yellow-5 (Tartrazine), Yellow-6, Annatto, Blue-1, Red 40, Carmine (cochineal extract or natural red-4)

* Source of information [4,5,6,7,8,9,10]; www.foodallergy.org/resources/food-additives-and-allergies/intolerances (assessed on 24 June 2023).

**Table 2:** Specific strains of probiotic species * are selectively used for their beneficial effects on gut health.

Genus	Species	Strain	Reference
*Bifidobacterium*	*infantis*	35624; Rossell-33	[39]
*Bifidobacterium*	*lactis*	Bi-07@; Bl-04@; HN019; BB-12@	[40,41,42,43,44]
*Bifidobacterium*	*breve*	M-16V@; Bbi99	[39,45]
*Bifidobacterium*	*animalis* ssp. *lactis*	BB-12^®^	[46,47,48]
*Bifidobacterium*	*bifidum*	Rosell-71	[49]
*Escherichia* strain	*coli*	Nissle 1917	[45]
*Bacillus*	*coagulans*	Unique IS-2; BC30^TM^	[39,50]
*Lactobacillus*	*reuteri*	Protectis@; RC-14@	[39]
*Lactobacillus*	*rhamnosus*	LGG@; HN001; GR-1@; Rosell-11; LC705	[39,51]
*Lactobacillus*	*plantarum*	DSM9843; LP299v@ (LP299v@)	[39,52]
*Lactobacillus*	*casei*	DN-114001@; Shirota@ DN001	[39]
*Lactobacillus*	*paracasei* ssp. *paracasei*	CASEI 431@; Lpc-37@	[46]
*Lactobacillus*	*acidophilus*	Rosell-52; NCFM@; LA05	[39,40,41,48]
*Saccharomyces*	*cerevisiae*	*boulardii*	[53,54,55]
*Propionibacterium*	*freudenreichii*	ssp. *shermanii* JS	[56]
*Lactobacillus*	*amylovorus*; *fermentum*		[57]
*Enterococcus*	*faecium*	SF68	[45]
*Lactobacillus*	*casei*	LOCK 0900; LOCK 0908;	[45]
*Lactobacillus*	*paracasei*	LOCK 0919	[45]
*Bacillus*	*Coagulans* (Ganeden BC30)	GBI-30, 6086	[58]

* Species names presented in the table are the same as in the corresponding references cited. The new nomenclature of some lactobacilli is as below: *Lactobacillus casei* as *Lacticaseibacillus casei*, *Lactobacillus reuteri* as *Limosilactobacillus reuteri, Lactobacillus rhamnosus* as *Lacticaseibacillus rhamnosus.* New nomenclature of other bacteria are available on https://isappscience.org/new-names-for-important-probiotic-lactobacillus-species/ (accessed on 4 June 2023).

**Table 3:** Probiotic cultures * are used for food and beverages fermenting dairy and non-dairy substrates.

Substrates/Product	Probiotic Culture	Strain	Reference
Cow’s milk/commercial dairy-based Probiotic beverage Kefir	*Bifidobacterium +* *Lactobacillus acidophilus +* *Lactobacillus casei +* *Lactobacillus rhamnosus +* *Lactobacillus plantarum*	Not disclosed	[73]
Oat, Coconut Cream, Rice Flour, Stabilisers (Tapioca Starch, Pectin)/commercial non-dairy-based Probiotic beverage Kefir	Live Vegan Kefir Cultures *Bifidobacterium*, *Lactobacillus acidophilus*, *Lactobacillus bulgaricus*, *Lactobacillus rhamnosus*	Not disclosed	[73]
Free Range Milk/Natural Bio-Yogurt	*Bifidobacterium animalis*, *Streptococcus thermophilus*, *Lactobacillus acidophillus*	BB12 is a particular strain of the *B. animalis*	[74]
Cow’s milk/commercial dairy-based Kefir Yogurt	*Bifidobacterium*, *Streptococcus thermophilus*, *Lactobacillus bulgaricus*, *Lactobacillus acidophilus*, *Lactobacillus casei*	Not disclosed	[73]
whole oats/a synbiotic food	*Lactobacillus plantarum*; *Bifidobacterium animalis*	*TK9*; subsp. *lactis V9*	[75,76]
Thai-pigmented rice/Novel probiotic products	*Bacillus coagulans*; *Lacti-caseibacillus rhamnosus*	*KPS-TF02*; *KPS-VE9*	[77]
Glutinous Rice/probiotic product	*Lactobacillus amylovorus*	*TISTR1110*	[78]
Rice, Oats and Inulin/Functional fermented food	*Lactobacillus rhamnosus*	*GR-1*	[79]
Formulations of germinated brown rice/fermented products functionalized by probiotics, with enhanced GABA, oryzanol and neutralized phytic acid	*Bifidobacterium longum*; *Bifidobacterium bifidum*; *Lacticaseibacillus rhamnosus*; *Streptococcus thermophilus*; *S. thermophilus + Lactobacillus del-brueckii* ssp. *Bulgaricus*; *Thermophilic LAB*	BB536; Bb-12; GG (ATCC 52103); Cryofast SST 31; Lyofast SY 1; YoFlex^®^YF-L02DA	[80]
Synbiotic Blend of Probiotic with Flaxseed	*Bacillus coagulans* (Ganeden BC30)	GBI-30, 6086	[58]
Functional fermented juice of a mixture of pineapple, spinach, cucumber, pumpkin, and Jerusalem artichoke juices	*Lacticaseibacillus rhamnosus*; *Lacticaseibacillus paracasei* subsp. *paracasei*; *Lactobacillus acidophilus*; *Bifidobacterium animalis* subsp. *lactis*; *Lactiplantibacillus plantarum*	n.a.	[81]
Oats, Barley and Malt/functional probiotic beverages	*Lactobacillus acidophilus*; *Lactobacillus plantarum*; *Lactobacillus reuteri*	*NCIMB 8821 NCIMB 8826 NCIMB 11951*	[82]
Corn-based/Functional beverage	*Lactobacillus paracasei*; *Saccharomyces cerevisiae*; *S. cerevisiae*; *Pichia kluyveri*	*LBC-81*; *CCMA 0731*; *CCMA 0732*; *CCMA 0615*	[83]
Cassava (Manihot esculenta Crantz) and rice-based/beverage with functional properties	*Lactobacillus plantarum*; *Torulaspora delbrueckii*; *Lactobacillus acidophilus*	*CCMA 0743*; *CCMA 0235*; *LAC-04*	[84]
Cassava and rice-based/beverage with functional properties	*Lactobacillus fermentum*; *Torulaspora delbrueckii*; *Pichia caribbica*; *Saccharomyces cerevisiae*	*CCMA 0215*; *CCMA 0234,0235*; *CCMA 0198*; *CCMA 0232*, *0233*	[85]
Maize blended with rice/Functional beverages	*Lactobacillus acidophilus*; *Lactobacillus plantarum*; *Torulaspora delbrueckii*	*LACA*; *CCMA 0743*; *CCMA 0235*	[86]
Rice-based fermented beverage “Bhaati Jaanr”	*Lactobacillus plantarum*	L7	[87]
Rice-based fermented beverage	*Lactobacillus fermentum*	KKL1	[88]
Quinoa beverage	*Lactobacillus plantarum*	DSM 9843	[89]
Soya-based fermented beverage	*Lactiplantibacillus plantarum*; *Lacticaseibacillus paracasei*	*CIDCA 8327*; *BGP1*	[90]

* Species names presented in the table are the same as in the corresponding references cited. The new nomenclature of some lactobacilli is as below: *Lactobacillus casei* as *Lacticaseibacillus casei*, *Lactobacillus reuteri* as *Limosilactobacillus reuteri*, *Lactobacillus rhamnosus* as *Lacticaseibacillus rhamnosus*. New nomenclature of other bacteria are available on https://isappscience.org/new-names-for-important-probiotic-lactobacillus-species/ (accessed on 4 June 2023).

## Data Availability

Not applicable.
